# A successfully shortened outbreak of vancomycin-resistant *Enterococcus faecium* in a secondary care hospital in Sweden

**DOI:** 10.1017/ash.2025.10251

**Published:** 2025-12-19

**Authors:** Maria Gideskog, Jenny Welander, Anita Hällgren, Lena Serrander

**Affiliations:** 1 Department of Communicable Disease and Infection Control, and Department of Biomedical and Clinical Sciences, Linköping University, Linköping, Sweden; 2 Department of Clinical Microbiology, and Department of Biomedical and Clinical Sciences, Linköping University, Linköping, Sweden; 3 Department of Infectious Diseases in Östergötland, and Department of Biomedical and Clinical Sciences, Linköping University, Linköping, Sweden

## Abstract

**Background:**

Outbreaks of vancomycin-resistant *Enterococcus faecium* (VRE) are often difficult to contain. In this study, we developed and implemented a set of control measures, which resulted in a relatively limited outbreak in a secondary care hospital in Sweden.

**Methods:**

VRE screening was performed by rapid polymerase chain reaction (PCR) on fecal swabs, reported within 1–3 h. Vancomycin-resistant isolates PCR-positive for the *vanA*/*vanB* gene were further analyzed with whole-genome sequencing (WGS). Cleaning efficiency was evaluated directly after cleaning by using adenosine triphosphate (ATP) swabs, detecting organic live material. The hospital management appointed a task force consisting of experts in infectious diseases, microbiology, hospital hygiene, cleaning and representatives of the affected unit.

**Results:**

A total of 22 VRE-positive patients were identified, of which 12 isolates belonged to the same clone (ST 203) in a surgical ward. VRE screening by PCR shortened the turnaround time. The combination of rapid PCR and WGS could rule in or out cases from the outbreak within less than a week. The new cleaning routine indicated that 3 approved quality-controlled discharge cleanings were required to reduce VRE acquisition. The fast lane to decision-making on control measures resulted in rapid introduction of the above routines.

**Conclusions:**

With prompt infection control measures, the VRE outbreak was contained after 4 months. To prevent further outbreaks of VRE, active rapid screening, improved cleaning, and restriction of multiple-bed rooms are efficient measures to implement.

## Background

Vancomycin-resistant *Enterococcus faecium* (VRE) has established itself as an important nosocomial pathogen. Infections caused by VRE include infective endocarditis, catheter-associated bloodstream infections, urinary tract infections, and bacteremia. Patients at risk for infection include those with critical illnesses in intensive care units, severe underlying disease, or a weakened immune system.^1,2^ VRE are hardy organisms with the ability to survive for long periods in adverse environmental conditions.^3^

Reducing VRE acquisition in healthcare environments require multiple strategies, including antimicrobial stewardship to reduce selection of VRE in colonized patients, appropriate infection control measures such as strict adherence to basic hygiene routines and adequate cleaning to reduce transmission, and reliable sensitive laboratory methods for detection of VRE in a timely manner.^1,4–6^ A previous study highlights the importance of identifying the source of VRE acquisition in the implementation of control measures, that is determining whether it is assumed to be due to cross-transmission or background acquisition.^7^ Polymerase chain reaction (PCR)-based screening has a high diagnostic accuracy and is, compared to culture-based screening, more timesaving in providing results. While culture turnaround time usually is several days, PCR results can be reported within just a few hours.^8^

The prevalence of VRE in Sweden remained low until 2007, when a large outbreak involving 3 hospitals in separate counties occurred.^9^ Since then, VRE outbreaks have been reported in several counties in Sweden. However, only sporadic, epidemiologically unrelated cases of VRE have been identified from clinical samples in Östergötland County. The aim of this study was to investigate a sudden increase in VRE cases in 2021 among patients admitted to a surgical unit at Vrinnevi Hospital, Norrköping, Östergötland County, Sweden, and to describe the measures implemented to contain the outbreak.

## Methods

### Settings

Vrinnevi Hospital is a secondary care hospital with 180 beds and a catchment population of approximately 200,000 inhabitants. The Surgical Unit at Vrinnevi Hospital comprised 2 wards at the time: ward 11, focusing on colorectal and acute general surgery, and ward 12, specializing in upper abdominal surgery and urology. Each ward offered 28 beds in 14 rooms (8 single-bed, 6 multi-bed: 4 × 4-bed and 2 × 2-bed rooms), with shared health care workers and medical supply storage.

The hospital’s infection prevention policy recommended single-bed rooms for VRE-positive patients, with VRE carriage consistently documented in patient records. However, no standardized screening protocol was in place prior to the outbreak, and ward design occasionally limited the availability of single rooms, as was the case in the Surgical Unit. Prior to the outbreak, adherence to basic hygiene routines ranged around 80%–90%, according to observation-based compliance audits.

An antibiotic audit and feedback program implemented at the Surgical Unit prior to the outbreak had reduced carbapenem and third-generation cephalosporin use in favor of piperacillin/tazobactam, which remained stable at the time of the outbreak. To further assess prescribing patterns, antibiotic prescriptions during the 3 months preceding the outbreak were reviewed. In addition, a meeting with the urological surgeons was held to discuss and implement updated antibiotic stewardship recommendations.

### Screening for VRE in patient and environmental samples

Patient and environmental samples were collected with ESwabs (Copan Diagnostics Inc. Murrieta, CA, USA). Screening for the *vanA*/*vanB* gene was performed directly from ESwabs using the GeneXpert system and the Xpert *vanA*/*vanB* kit (Cepheid, Sunnyvale, CA, USA), following the manufacturer’s instructions. Fecal samples were collected from patients who had either been transferred from the Surgical Unit at Vrinnevi Hospital to another unit or readmitted to the hospital after previously receiving care at the Surgical Unit.

As a complement to the individual rapid screening, weekly batched screening was performed of all patients at the Surgical Unit by direct DNA extraction from ESwabs using the STARMag Viral DNA/RNA 200C kit on the Seegene STARlet IVD system, followed by real-time PCR with the Allplex™ Entero-DR Assay (Seegene Inc., Seoul, South Korea) on the CFX96™ real-time PCR detection system (Bio-Rad, Hercules, CA, USA). All methods were carried out in accordance with the manufacturer’s instructions. The same screening approach was applied to units with patient exchange with the Surgical Unit. Samples testing positive for *van* genes were subsequently cultured for confirmation, as described below. The screening protocol included continued sampling until 1 month had passed without detection of new VRE cases before declaring the outbreak contained. An outbreak was defined as the occurrence of more than 2 epidemiologically related cases.

Environmental samples were inoculated into VRE broth supplemented with vancomycin (0.75 mg/L, Sigma-Aldrich, St. Louis, MO, USA) and aztreonam (10 mg/L, MP Biomedicals, Illkirch-Graffenstaden, France) overnight and thereafter onto VRE Chromagar plates supplemented with chloramphenicol and dipotassium telluride (CHROMagar, Paris, France). The plates were incubated at 35°C for 48 h. Bacteria were identified to the species level with a MALDI Biotyper 3.0 (Bruker Corporation, Karlsruhe, Germany). *E. faecium* isolates with resistance to vancomycin were subjected to GeneXpert for verification of the *vanA* or *vanB* gene.

The preliminary reporting times for each method were as follows: 1–3 h for GeneXpert, 1 day for Seegene, and the final report for all samples was completed within 3 days.

### WGS

All VRE isolates from patients and the environment were subjected to whole-genome sequencing (WGS). DNA was prepared from 10 µL of each isolate using an EZ1 DNA Tissue Kit (Qiagen, Germantown, MD, USA) with an included preheating step at 95°C and shaking at 350 rpm. Twenty nanograms of DNA was used for library preparation with a QIAseq FX DNA Library Kit (Qiagen, Germantown, MD, USA) with 8 min of fragmentation time. DNA libraries were sequenced on the MiSeq platform (Illumina, San Diego, CA, USA) with 2 × 300 bp paired-end reads, and an average sequencing depth of 210x was achieved for the samples.

Data analysis was performed in CLC Genomics Workbench v. 10.1.1 with the Microbial Genomics Module v. 2.5.1 (Qiagen, Germantown, MD, USA). Multilocus sequence typing (MLST) analysis was performed using the PubMLST (pubmlst.org)^10^ scheme for *Enterococcus faecium.* ^11^ Read mapping and variant calling were performed against the *E. faecium* reference genome with NCBI accession number NC_021994, with the following thresholds to call a variant: depth of coverage ≥ 20, frequency ≥ 90 % and Phred score ≥ 20. A quality filter was then applied that retained variants with a sequencing depth of ≥ 20x in all samples and a distance ≥ 10 bp to the next variant, and the resulting variants were used to create a single nucleotide polymorphism (SNP) tree and calculate genetic distances between samples. Previous studies suggest that isolates of *E. faecium* with a distance of ≤16 SNPs are likely to belong to the same clone.^12^

### Cleaning and ATP measurements

Initially, rooms that had been occupied by confirmed VRE-positive patients were discharge cleaned once using Virkon™ (1%) and an alcohol-based disinfectant containing a surfactant. To assess environmental contamination and cleaning efficacy, samples were collected from high-touch surfaces in ward 12 previously occupied by VRE-positive patients. Since environmental cultures remained positive, triggering new episodes of VRE transmission, the discharge cleaning, including medical devises, was intensified to 3 times followed by visual hygiene observation and an approved quality control in terms of adenosine triphosphate (ATP) measurement to detect organic live material according to the manufacturer’s instructions (Neogen, Lansing, MI, USA). Cleaning followed routine procedures, with all surfaces cleaned, not only contaminated ones. Twenty locations in each patient room, including the bathroom, were subjects for ATP measurement, that is a total of 60 locations were measured in each discharge-cleaned patient room. All additional patient rooms at the Surgical Unit (non-VRE) were discharge-cleaned once with a subsequent approved quality control. Even the daily cleaning was quality-controlled, and disinfection of general surfaces was extended. During this process, the outbreak task force actively ensured that cleaning products were reviewed for efficacy, proper contact times were adhered to, cleaning workflows were observed, and responsibilities for cleaning were clearly defined. In connection with the intensified cleaning routine, the cleaning organization temporarily took over cleaning of patient rooms, including the daily cleaning of surfaces close to the patient, from healthcare workers for the remainder of the outbreak.

## Results

### Outbreak overview

**Figure 1.**
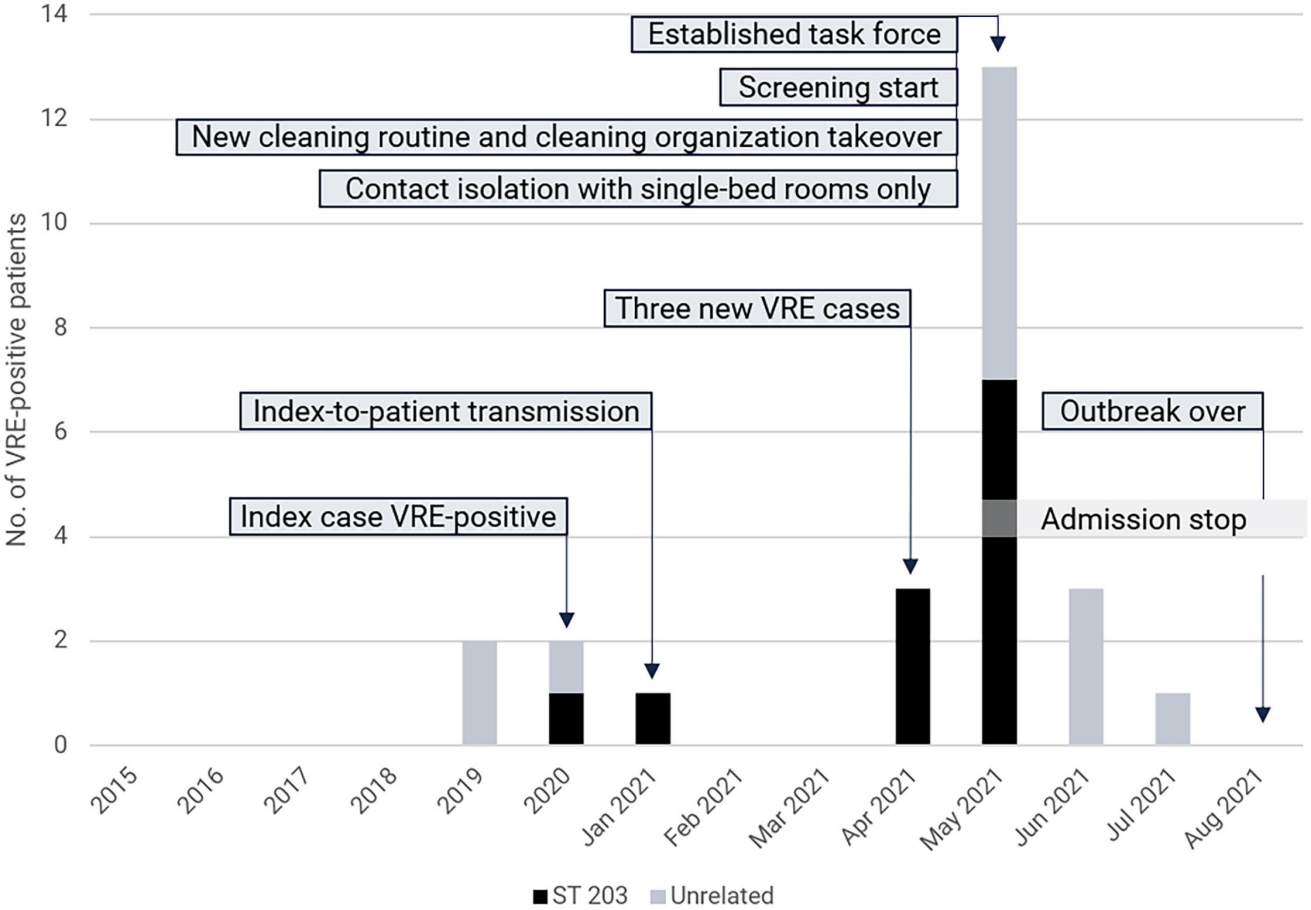
Prevalence of patients positive for vancomycin-resistant *enterococcus faecium* (VRE) at Vrinnevi Hospital, Norrköping, Sweden, 2015—August 2021. Bars indicate the total number of VRE-positive cases identified each year. Outbreak-associated cases (ST 203) and unrelated cases are shown in distinct colors. Infection control measures implemented during the outbreak are indicated on the time line.

In April 2021, a VRE-positive patient (index case), identified from a clinical sample, was re-admitted and placed in a single-bed room (room 14) in ward 12 at the Surgical Unit at Vrinnevi Hospital. The patient had been admitted to the ward on several previous occasions and placed in different single- and 4-bed rooms. A few months earlier, VRE was transmitted from the index case to another patient while placed in different rooms in ward 12. The transmitted *E. faecium* strain carried the *vanA* gene. Three additional VRE cases were identified through clinical cultures within a week of the index case’s re-admission to ward 12 (Figure 1). These patients were placed in 2 different 4-bed rooms with a shared bathroom in ward 12, including room 9, which had previously been occupied by the index case in the same bed space. To quickly and effectively minimize the extent of the VRE outbreak, the hospital management appointed a task force with experts in infectious diseases, microbiology, hospital hygiene, cleaning and representatives of the affected unit that had weekly meetings to discuss the current situation and operatively handle the outbreak.

Subsequent screening for VRE was initiated as described above. Cases were defined as patients colonized with VRE, with the same *van* gene as the index case. An additional inclusion criterion was that there had to be a connection in time and space with the index case or another VRE-positive patient involved in the outbreak. Approximately 800 patients were screened for VRE during the screening period, which lasted from the end of April until the end of August 2021. The last VRE case occurred in July, and after 1 month without new cases, the outbreak was declared contained. Eight more cases were reported in ward 12 until the end of May. Additional VRE cases were reported at the Geriatric Unit (*n* = 2), the Emergency Unit (*n* = 2), ward 11 at the Surgical Unit (*n* = 1), the Cardiology Unit (*n* = 1), and the Infectious Diseases Unit (*n* = 1) at Vrinnevi Hospital, and 1 case was reported in a senior center in Norrköping. Another case was admitted to the Haematology Unit at Linköping University Hospital. The majority had previously received multiple antibiotic treatments, as documented in the medical record.

Hygiene observations by the Department of Communicable Disease and Infection Control revealed several deficiencies at the Surgical Unit. These were promptly addressed through control measures, including education and review of proper procedures to improve compliance with basic hygiene and cleaning routines, replacement of furniture and equipment that were impossible to disinfect, and clearing surfaces to enable effective disinfection. Patient-dedicated equipment was allocated. An admission stop and contact isolation were introduced in ward 12, with all multiple-bed rooms converted to single-bed rooms. Adherence to basic hygiene routines was observed to improve by the later stages of the outbreak and afterward, reaching consistently high levels of around 90%.

The review of antibiotic prescriptions from the 3 months preceding the outbreak showed that piperacillin/tazobactam accounted for over 50% of all prescriptions, while oral cephalosporins and ciprofloxacin dominated the remaining use. Updated antibiotic stewardship recommendations were subsequently implemented.

Beyond clinical and organizational measures, the outbreak also had substantial economic consequences. The estimated cost of the outbreak response was approximately 2.6 million SEK.

### WGS results

**Figure 2.**
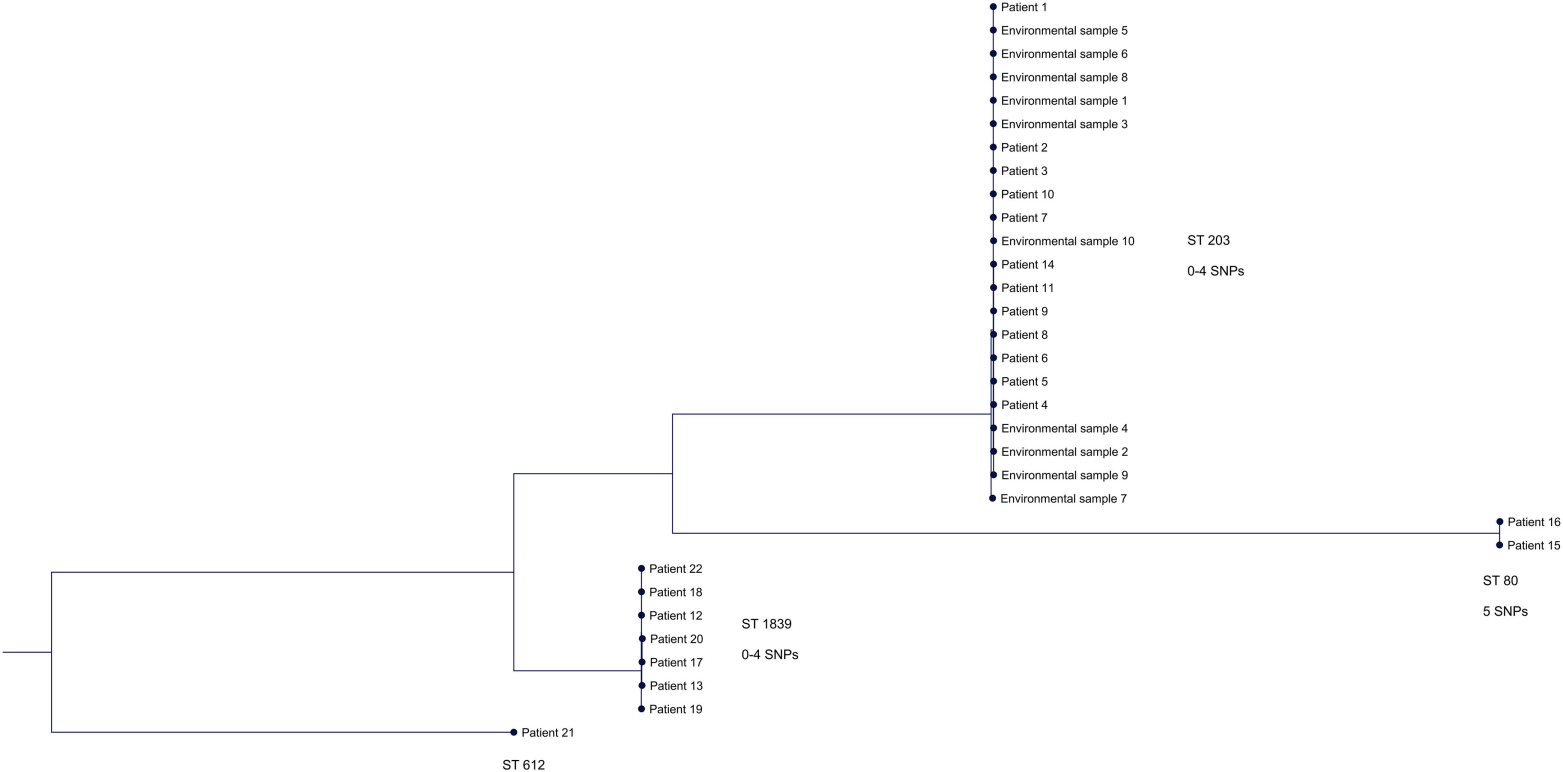
Phylogenetic tree based on single nucleotide polymorphism (SNP) analysis of whole-genome sequencing (WGS) data from 32 vancomycin-resistant *enterococcus faecium* (VRE) isolates. Four outbreak clones were identified: ST 203 (isolates differing by 0–4 SNPs), ST 1839 (isolates differing by 0–4 SNPs), ST 80 (isolates differing by 5 SNP), and ST 612 (unique clone).

**Figure 3.**
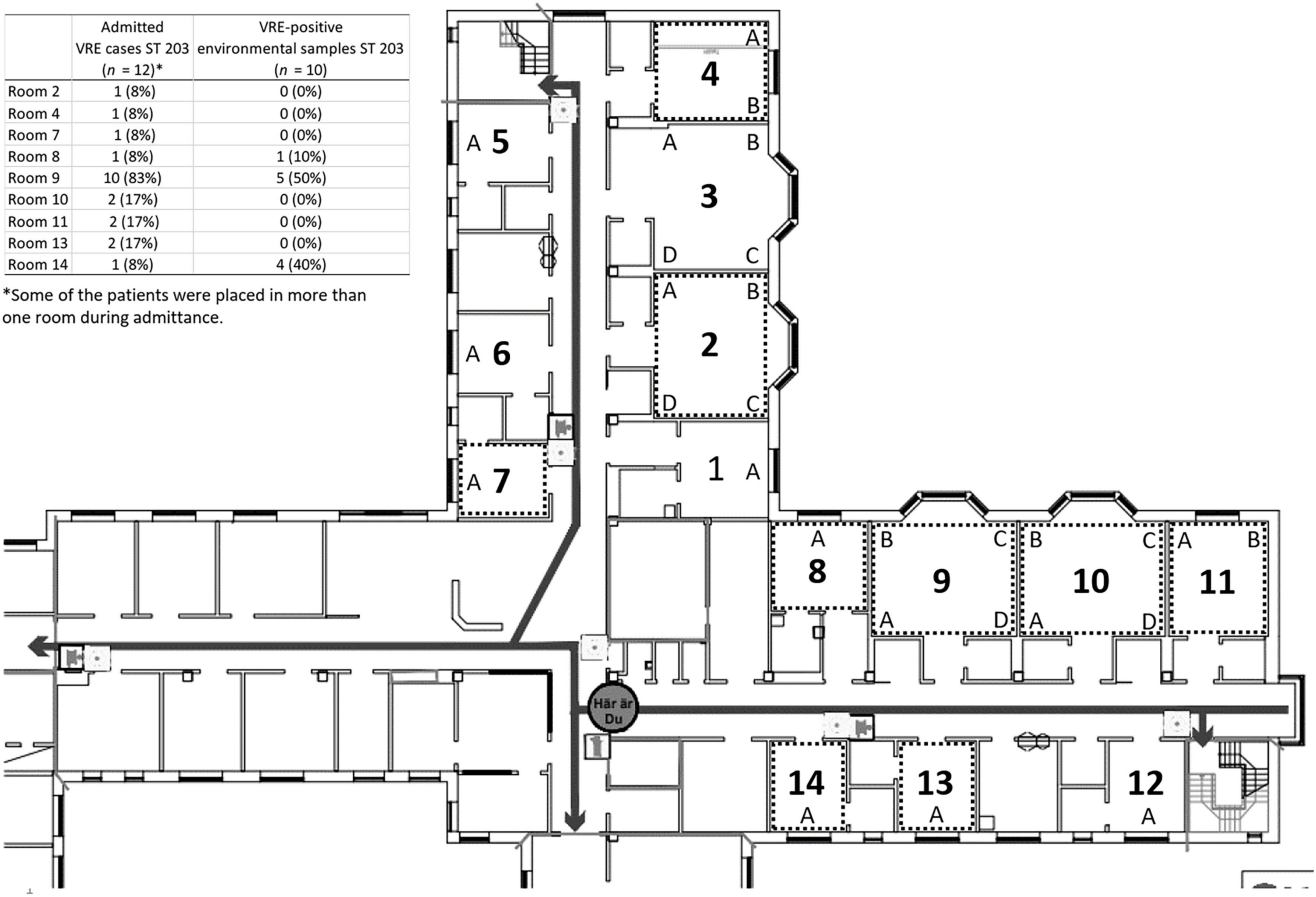
Drawing of ward 12 at the Surgical Unit at Vrinnevi Hospital, Norrköping, Sweden, where the clustering of vancomycin-resistant *enterococcus faecium* (VRE) took place. Letters (A–D) indicate the number of available bed spaces per room. Rooms where patients culture-positive for the ST 203 clone had been placed are marked with dotted squares. The numbers of admitted VRE cases and VRE-positive environmental samples per room are shown in the inserted table.

Three outbreak clusters and 1 unique clone were recognized with MLST and whole genome-wide phylogenetic analysis (Figure 2). The larger cluster consisted of 22 isolates (69%) with a difference of 0–4 SNPs and belonged to the ST 203 clone. Included in this cluster were 12 VRE cases reported in ward 12 and all 10 VRE-positive environmental samples. Of these patients, 83% had been accommodated in the same 4-bed room (room 9) at some time during their admission (Figure 3). In the second cluster, 7 isolates (22%) with a difference of 0–4 SNPs and that belonged to ST 1839 were included. Among these isolates were 2 VRE cases reported in the Geriatric Unit, 2 in the Emergency Unit, 1 in ward 12, 1 in the Cardiology Unit, and 1 in a senior center in Norrköping. The third cluster consisted of 2 isolates (6%) of ST 80 with a difference of 5 SNPs from a patient in the Emergency Unit and another patient admitted to ward 11 at the Surgical Unit at Vrinnevi Hospital. The unique clone (ST 612) belonged to a patient admitted to the Haematology Unit at Linköping University Hospital (3%).

### Evaluation of cleaning procedures

Of the 242 environmental samples collected before the improved cleaning in ward 12, 10 (4,1%) were positive for the ST 203 outbreak clone, which shows that the VRE acquisition was not sufficiently reduced. Nine samples were found either in the single-bed room (room 14), where the index case had recently been placed (40%), or in the 4-bed room (room 9), previously occupied by VRE-positive patients (50 %) (Figure 3). The culture-positive sites included general surfaces in patient rooms and bathrooms, such as the toilet handle, seat, and roll holder, as well as the bed rail, mattress, armchair, and the NEWS (National Early Warning System) tool. Most positive samples originated from the bathroom. After implementing 3 approved quality-controlled discharge cleanings at the Surgical Unit, none of the collected environmental samples (*n* = 104) in ward 12 showed growth of VRE. The direct feedback on whether approved ATP values had been achieved after each cleaning resulted in higher quality of cleaning, which was apparently sufficient in reducing the VRE acquisition. The cleaning routine was implemented across all hospital units in Östergötland County with VRE-positive patients.

## Discussion

In the present study, the first VRE outbreak in Östergötland County, Sweden, at the Surgical Unit of Vrinnevi Hospital in Norrköping, was described. Active rapid screening was initiated and provided important real-time data to direct actions to reduce the outbreak. By using PCR directly from fecal swabs, the delay inherent to culture-based methods, often requiring several days for bacterial growth and identification, was effectively eliminated and allowed for much faster detection of carriers. Early detection of VRE carriers is crucial in limiting transmission and plays a key role in effective outbreak management as described in other studies.^7,8,13^

This is one of the first studies to use WGS during an outbreak rather than retrospectively to quickly determine whether isolates are related. The use of WGS and subsequent MLST analysis was crucial in clarifying the outbreak dynamics. It enabled rapid assessment of the genetic relatedness between VRE isolates and allowed assignment to specific clusters. Among the 22 patients involved, the main outbreak clone (ST203) was identified in ward 12 at the Surgical Unit, distinct from the three other clones detected during the same period. Studies have shown an increase in VRE colonization during hospital stay, and that patients sharing a room and a toilet with a VRE carrier had a high acquisition rate.^1,14^ This aligns with our findings of high proportions of VRE cases and positive environmental samples in ward 12, particularly in room 9, suggesting that patient proximity and shared facilities facilitated transmission.

Although lack of compliance to basic hygiene routines is one of the main reasons why hospital outbreaks occur, previous studies have highlighted rapid identification of cases and adequate cleaning as important factors in controlling VRE outbreaks.^15^ The cleaning routine implemented in the Surgical Unit, comprising 3 approved discharge cleanings per room after a VRE-positive patient, proved effective and resulted in a drastic reduction in both VRE cases and environmental contamination. This aligns with a previous study showing that repeated cleaning improves VRE decontamination.^16^ ATP measurement serves as an effective quantitative method to assess cleaning quality in healthcare settings, aiding in the detection of residual contamination after disinfection^17,18^ A major challenge during the outbreak was confusion over whether healthcare workers in the unit or the cleaning organization were responsible for cleaning certain environmental surfaces and medical devices. Additionally, executors lacked knowledge about proper cleaning frequency and methods, potentially leading to inadequate disinfection. Similar concerns have been reported in other studies.^19–21^

Antibiotic exposure, especially to substances with a high likelihood of disturbing the gut flora, has been identified as a risk factor for both the acquisition and transmission of VRE, and consequently, antibiotic stewardship is of importance both to prevent and confine an outbreak of VRE.^22–24^ The present outbreak, although the main efforts focused on hygiene measures, highlighted that antibiotic prescribing related to urological surgery needed improvement, and measures were implemented accordingly.

A limitation of this study is that multiple interventions were implemented simultaneously, making it difficult to determine the relative contribution and effectiveness of each measure in controlling the outbreak.

## Conclusions

An outbreak of VRE involving 22 patients and 4 distinct clones was confirmed with WGS. An empowered task force, active rapid screening, timely WGS typing, and quality-controlled cleaning were effective. To prevent further outbreaks, antibiotic stewardship was strengthened, a 3-step quality-controlled discharge cleaning routine maintained, and use of multiple-bed rooms restricted.
